# Palliative effect of taurine against hepatic injury induced by polystyrene microplastics through antioxidant and metabolic pathway modulation in mice

**DOI:** 10.3389/fphar.2025.1665161

**Published:** 2025-10-02

**Authors:** Areej A. Eskandrani, Amany Abdel-Rahman Mohamed, Badriyah S. Alotaibi, Yasmina M. Abd El-Hakim, Tarek Khamis, Ahmed E. Noreldin, Ahmed E Abdelhamid, Nawal Alsubaie, Leena S. Alqahtani

**Affiliations:** ^1^ Chemistry Department, College of Science, Taibah University, Medina, Saudi Arabia; ^2^ Department of Forensic Medicine and Toxicology, Faculty of Veterinary Medicine, Zagazig University, Zagazig, Egypt; ^3^ Department of Pharmaceutical Sciences, College of Pharmacy, Princess Nourah bint Abdulrahman University, Riyadh, Saudi Arabia; ^4^ Pharmacology Department, Faculty of Veterinary Medicine, Zagazig University, Zagazig, Egypt; ^5^ Laboratory of Biotechnology, Faculty of Veterinary Medicine, Zagazig University, Zagazig, Egypt; ^6^ Histology and Cytology Department, Faculty of Veterinary Medicine, Damanhour University, Damanhour, Egypt; ^7^ Polymers and Pigments Department, National Research Centre, Giza, Egypt; ^8^ Department of Pharmacy Practice, College of Pharmacy, Princess Nourah bint Abdulrahman University, Riyadh, Saudi Arabia; ^9^ Department of Biological Sciences, College of Science, University of Jeddah, Jeddah, Saudi Arabia

**Keywords:** liver, gene expression, microplastics, oxidative stress, taurine, lipid metabolic disturbances, histological assessments

## Abstract

**Introduction:**

Microplastics (MPs), particularly polystyrene microplastics (PS-MPs), are emerging environmental contaminants that have been shown to accumulate in various organs, including the liver, leading to oxidative stress, inflammation, and metabolic dysregulation. However, the precise molecular mechanisms underlying PS-MP-induced hepatotoxicity and disruptions in lipid metabolism remain poorly understood. Taurine (Tau), a naturally occurring amino acid with known antioxidant and cytoprotective properties, may suggest a potential protective strategy against such toxicity. This study aimed to investigate the hepatotoxic effects of PS-MPs in a mouse model and to evaluate the potential ameliorative role of Tau.

**Methods:**

Mice were exposed to PS-MPs with or without Tau supplementation over a 60-day experimental period. The groups were: control group, which received distilled water orally (0.5 mL/mouse). The Tau group was administered Tau at a dose of 200 mg/kg body weight. The PS-MPs group received PS-MPs at 10 mg/kg body weight, suspended in distilled water. The combination group (PS-MPs + Tau) received both Tau and PS-MPs at the same doses concurrently. Multiple endpoints were assessed, including oxidative stress biomarkers, liver function indicators, lipid and bilirubin profiles, histopathological alterations, and the expression of key genes involved in lipid metabolism and oxidative stress regulation.

**Results:**

Exposure to PS-MPs resulted in notable hepatic injury, characterized by elevated oxidative stress, dysregulated lipid profiles, impaired antioxidant enzyme activity, and altered expression of genes related to lipogenesis and fatty acid oxidation compared to the control. Histological examination revealed congested central and portal veins, massive aggregations of lymphocytes, the hepatocytes appeared markedly swollen, disorganized arrangement, and exhibited large nuclei with strong basophilic staining consistent with these biochemical findings. Co-administration of Tau mitigated these adverse effects, improving antioxidant status, normalizing metabolic markers, and partially restoring gene expression patterns and tissue integrity.

**Conclusion:**

Overall, the findings indicated that PS-MPs caused liver damage via oxidative stress and lipid metabolic disturbance, and that Tau supplementation had a protective effect, possibly via modulating oxidative and metabolic pathways. This experiment emphasized the necessity for additional research into Tau as a therapeutic agent in microplastic-related liver damage.

## 1 Introduction

In recent years, plastic pollution has become a pervasive environmental concern, with plastic products making up a substantial portion of marine debris due to their widespread use across industries and in everyday life (MacLeod et al., 2021). Over time, these plastics degrade through physical, chemical, and biological processes, resulting in the formation of microplastics (MPs), defined as plastic particles less than 5 mm in size (Ahmad et al., 2023). MPs have been widely detected across various ecosystems, including freshwater and marine environments, sediments, urban beaches, and wastewater systems (Bellasi et al., 2020). Due to their small size and resistance to degradation, MPs can be easily ingested and accumulate in the tissues of both aquatic and terrestrial organisms (Lin et al., 2023).

The increasing detection of MPs in human biological samples such as stool, placenta, and even lung tissues highlights growing concerns about their potential health impacts (Ragusa et al., 2021; Schwabl et al., 2019). PS-MPs, in particular, are among the most prevalent synthetic particles found in polluted environments (Yaseen et al., 2022). Several studies have reported that ingestion or inhalation of PS-MPs can lead to significant biological disturbances, including disruptions in gut microbiota (Qiao et al., 2021), liver metabolic dysfunction (Yin et al., 2022), renal damage (Wang et al., 2021), and neurotoxicity (Prüst et al., 2020).

Oxidative stress is one of the key mechanisms through which MPs exert toxic effects, resulting from an imbalance between reactive oxygen species (ROS) production and the body’s antioxidant defences (Jabeen et al., 2023). Excessive ROS can cause widespread cellular damage, including lipid peroxidation, protein denaturation, DNA mutations, and ultimately cell death through apoptosis or necrosis (Ehsan et al., 2023; Mostafa-Hedeab et al., 2023). The liver, as a central organ for detoxification and metabolism, is particularly vulnerable due to its high mitochondrial density, a major site of ROS generation (Mahfouz et al., 2023). Recent studies propose that oxidative stress is a key pathogenic mechanism in microplastic-induced hepatotoxicity (Blackburn and Green, 2022; Cheng et al., 2022). Studies using animal models such as mice and zebrafish have demonstrated that MPs can cross the intestinal barrier and subsequently accumulate in the liver, likely via translocation through the bloodstream (Ašmonaitė et al., 2018; Liu P. et al., 2020; Liu S. et al., 2020; Rubio et al., 2020). Exposure to PS-MPs, whether 5 μm or 70 nm in size, has been shown to trigger oxidative stress and inflammatory responses (Ijaz et al., 2024). Specifically, zebrafish (Pei et al., 2022) and mice (Zou et al., 2023) exposed to these particles displayed declined activities of antioxidant enzymes like catalase and superoxide dismutase (SOD), along with elevated malondialdehyde (MDA) levels and augmented expression of pro-inflammatory cytokines, such as TNF-α and IL-1β (Li et al., 2021; Lu et al., 2016). Moreover, microplastic exposure has been linked to hepatic steatosis and altered lipid metabolism in the liver of both species, indicating a typical profile of hepatotoxicity (Luo et al., 2019; Zheng et al., 2021).

Recent evidence indicates that microplastics, depending on their size and composition, may differentially affect liver function (Hu and Palić, 2020) Given the liver’s central role in systemic homeostasis and its susceptibility to microplastic accumulation, further investigation into the molecular mechanisms of PS-MP-induced hepatotoxicity is critical.

Taurine (Tau) is a sulfur-containing, conditionally essential amino acid that is among the most abundant in mammalian tissues, with high intracellular concentrations in various cell types, including hepatocytes (Lambert et al., 2015). It is both synthesized in the liver and acquired through the diet, and has a key role in a wide range of physiological processes such as bile acid conjugation, osmoregulation, membrane stabilization, calcium signalling, and detoxification of xenobiotics (Schaffer et al., 2000). Furthermore, dietary supplementation of Tau has been associated with a decreased hazard of several diseases, including diabetes (Piao et al., 2018), metabolic syndrome (Chen et al., 2016), skeletal muscle problems (De Luca et al., 2015), liver disease (Miyazaki and Matsuzaki, 2014), and central nervous system disorders (Menzie et al., 2014).

In the context of liver health, Tau has demonstrated potent antioxidant and anti-inflammatory properties. It scavenges reactive oxygen species (ROS) both directly and indirectly by increasing the activity of natural antioxidant enzymes such as superoxide dismutase (SOD), catalase (CAT), and glutathione peroxidase (GPx) (de Oliveira Ramos et al., 2018). Additionally, Tau contributes to maintaining mitochondrial integrity and reducing lipid peroxidation (Zhang et al., 2014). The anti-inflammatory effect is achieved via inhibiting pro-inflammatory cytokines such as TNF-α and IL-1β, as well as suppressing NF-κB activation, a central pathway in inflammation and immune activation in CCL4-induced hepatic damage (Abdel-Moneim et al., 2015). Tau chloramine, a derivative formed during immune responses, is known to modulate inflammation by downregulating excessive immune activity (Qaradakhi et al., 2020). Despite these established roles, the precise molecular targets of Tau *in vivo* remain under investigation, which has so far limited its full clinical application (Schaffer and Kim, 2018). Nonetheless, its multifaceted protective mechanisms make Tau a promising candidate for alleviating liver injury, particularly that induced by environmental pollutants such as microplastics.

Although the environmental and biological risks of MP exposure are increasingly recognized, our understanding of the underlying mechanisms of MP-induced organ toxicity, particularly hepatotoxicity, remains limited. PS-MPs, among the most common types of MPs, have been shown to accumulate in liver tissue and induce oxidative stress, inflammation, and metabolic disturbances in animal models. However, the specific impact of PS-MPs on hepatic lipid metabolism and liver function at the molecular level is still not well defined. Furthermore, there is a lack of effective mitigation strategies to counteract these toxic effects. Tau, a naturally occurring amino acid with known antioxidant properties, may represent a promising intervention, but its role in protecting against microplastic-induced liver injury has not yet been fully explored. Therefore, the present study was designed to investigate the hepatotoxic effects of PS-MPs in a mouse model by assessing oxidative stress biomarkers, lipid and bilirubin profiles, histopathological changes, and gene expression related to hepatic lipid metabolism. Additionally, we aim to evaluate the potential ameliorative effect of Tau in reducing PS-MP-induced liver damage. We hypothesize that PS-MP exposure leads to liver toxicity through oxidative stress and metabolic disruption, and that Tau supplementation can attenuate these effects and restore hepatic homeostasis.

## 2 Materials and methods

### 2.1 Preparation and characterization of PS-MP

PS-MPs were synthesized via the suspension polymerization method, as previously described in the literature (Cho et al., 2016). In brief, 50 mL of styrene monomer was mixed with 0.5 g of benzoyl peroxide (used as an initiator), and the mixture was added to 200 mL of ethanol containing 2 g of polyvinylpyrrolidone (PVP, MW: 40 kDa), which served as a stabilizer. The reaction mixture was continuously stirred and maintained at 70 °C in an oil bath equipped with a condenser for approximately 24 h. During the polymerization process, the formation of a white suspension indicated the generation of polystyrene microbeads. Upon completion of the reaction, the PS-MPs were collected by centrifugation at 5,000 rpm, washed successively with distilled water and ethanol to remove unreacted materials, and subsequently air-dried at room temperature to a constant weight.

The chemical composition and functional groups of the synthesized PS-MPs were analyzed using Attenuated Total Reflection Fourier Transform Infrared Spectroscopy (ATR-FTIR) with an ALPHA-II FTIR spectrometer (Bruker Optik GmbH, Germany), operating over a spectral range of 4,000–400 cm^-1^ at a resolution of 4 cm^-1^ (Fang et al., 2010). Morphological characteristics and particle size were examined using a scanning electron microscope (QUANTA FEG 250, ESEM) under an accelerating voltage ranging from 200 V to 30 kV (Karanam et al., 2024). The hydrodynamic diameter of the PS-MPs was further measured using dynamic light scattering (DLS) analysis with a Zetasizer Nano-ZS (Malvern Instruments Ltd., UK, Zetasizer version 7.04).

### 2.2 Tested compound tau

Tau was purchased from Alfa Chemistry Co. (101–5 Colin Dr, Holbrook, NY 11741). Purity 98%, Catalog Number (ACM107357-5).

### 2.3 Animals and experimental design

Eighty healthy and pathogen-free Swiss male mice (20–25 g body weight, 6 weeks old) were procured from the Laboratory Animal Housing Unit at the Faculty of Veterinary Medicine at Zagazig University in Egypt. Mice were housed in stainless steel cages with free access to food and water in a well-ventilated room with a light/dark cycle of 12 h each day. Before using in any of the experiments described here, the experimental animals were adapted to the laboratory setting for 14 days pre-dosing.

### 2.4 Experimental scheme

Eighty Swiss mice were randomly divided into four groups (n = 20/group). The control group received distilled water orally (0.5 mL/mouse). The Tau group was administered taurine (Tau) at a dose of 200 mg/kg body weight, following previously well-known protocols (Ma et al., 2021; Song et al., 2024). The Ps-MPs group received Ps-MPs at 10 mg/kg body weight, prepared in distilled water; the volume administered was calculated using the formula: Volume (L) = Required dose (mg/kg)/Concentration of solution (mg/L). The combination group (PS-MPs + Tau) received both treatments at the same respective doses for 60 consecutive days. The tested compounds, including PS-MPs and Tau, were administered orally by gavage using a feeding needle to ensure accurate and consistent dosing. In the combination group, PS-MPs and taurine were administered with a 1-h interval between the two substances to guarantee precise dosage delivery and to minimize the possibility of direct interaction. Mice were weighed weekly to adjust doses based on weight changes, and they were observed daily for signs of distress, abnormal behaviour, respiratory abnormalities, mucous membrane colour changes, sickness, or death.

### 2.5 Tau dose selection

A dose of 200 mg/kg body weight (bwt) of Tau was selected based on prior experimental evidence demonstrating its efficacy in modulating oxidative stress, inflammation, and apoptosis in murine models (Niu et al., 2018). Specifically, the dose, corresponding to approximately 200 μmol/kg, has been previously reported to exhibit significant antioxidant, anti-inflammatory, and anti-apoptotic properties, particularly in the context of cardiovascular protection (Song et al., 2024). Based on this, we hypothesized that Tau at this effective dose may also confer hepatoprotective effects, particularly against oxidative stress, hepatocellular damage, and inflammation induced by PS-MPs exposure.

### 2.6 PS-MPs dose selection

Oral gavage was selected as the mode of administration, reflecting ingestion as the primary route of microplastic exposure in humans (Allouzi et al., 2021; Campanale et al., 2020). The administered dose of 10 mg/kg body weight (equivalent to roughly 0.6 g for a 60 kg adult) falls within the estimated human intake range of microplastics, reported to be between 0.1 and 5 g per week (Senathirajah et al., 2021).

### 2.7 Detection of hepatosomatic index

Hepatosomatic index (HSI) was calculated to assess relative liver size and potential hepatomegaly. The livers were carefully excised, cleaned of adherent fat and connective tissue, and weighed using a digital analytical balance with 0.01 g precision. The HSI was determined using the following formula: [Liver weight (g)/body weight (g) ]x 100.

Where:

• Liver weight (g) is the absolute wet weight of the liver immediately after dissection.
• Body weight (g) is the final body weight of the mouse recorded prior to sacrifice.

### 2.8 Sample collection

At the end of the exposure period, mice were weighed, and isoflurane was administered in an induction chamber until loss of consciousness, then cervical dislocation was applied. Blood samples were collected from the orbital sinus without an anticoagulant, and serum was separated by centrifugation at 3,000 rpm for 10 min. The serum was stored at −20 °C for subsequent biochemical analyses, including liver enzymes, lipid profile, and bilirubin levels. Liver tissues were carefully excised, rinsed with ice-cold saline to remove blood residues, and divided into three portions for comprehensive analysis. The first portion was homogenized at 4 °C (15 min at 664 × g) for the assessment of antioxidant enzyme activities and lipid peroxidation levels (MDA). The second portion was fixed in 10% neutral-buffered formalin for histopathological evaluation. The third portion was preserved at −80 °C for total RNA extraction and subsequent quantitative real-time PCR (qRT-PCR) to assess gene expression.

### 2.9 Evaluation of hepatocellular enzymes

Serum levels of aspartate aminotransferase (AST) and alanine aminotransferase (ALT) were determined using colorimetric commercial kits (Sigma-Aldrich, Cat. No. Cat. No. MAK467 and MAK052) based on the spectrophotometric method described by Reitman and Frankel (1957). Absorbance was read using a microplate reader (Bio-Tek Instruments, Winooski, VT, USA). Also, Gamma glutamyl transferase (GGT) was diagnosed via colorimetric kits of My BioSource (San Diego, USA), catalogue no. MBS165115.

### 2.10 Estimation of bilirubin content, lipid profile, and total protein content

Bilirubin content, serum triglycerides, and serum cholesterol were detected in the sera of all experimental groups via the methods of Walters and Gerarde (1970), Foster and Dunn (1973), and Zlatkis et al. (1953), respectively, using a biochemistry analyzer system (Synchron Clinical System Lx 20; Beckman Coulter Inc., Fullerton, CA, USA). Additionally, the low-density lipoprotein cholesterol (LDL-C) fraction was estimated using the formula of Friedewald et al. (1972).

$$\text{LDL}-\text{cholesterol}=\text{Total cholesterol }–\;\left(\text{Triglyceride}/5+\text{HDL}-\text{ cholesterol}\right)$$

### 2.11 Evaluation of the liver homogenate malondialdehyde and antioxidants (MDA)

Each frozen liver tissue sample was homogenized in an ice bath for 10 min using a Polytron PT one200 E homogenizer with 10 volumes of a 50 mM Tris buffer containing 10 mM EDTA (pH 7.5). The homogenate was then centrifuged at 1,000 × g for 10 min at 4 °C. The homogenate was used for the assessment of oxidative stress markers. Malondialdehyde (MDA) levels were determined using the Thiobarbituric Acid Reactive Substances (TBARS) assay, Ohkawa et al. (1979).

The superoxide dismutase (SOD) activities were detected using Bio-diagnostic colorimetric kits (Giza, Egypt) with Catalogue numbers SD 25 21. EnzyChromTM GSH/GSSG Assay Kit (EGTT-100) obtained from BioAssay Systems (Hayward, CA, USA) was selected to determine the GSH/GSSG ratio. Catalase (CAT) levels were estimated in our experiment using Bio-diagnostic Co., Egypt kits, based on the protocol described by Beutler et al. (1963).

### 2.12 Gene expression

The expression of several key hepatic genes *AMPK-1* (AMP-activated protein kinase alpha-1), *ACC* (acetyl-CoA carboxylase), CPT-1 (carnitine palmitoyltransferase-1), *SREBP-1* (sterol regulatory element-binding protein-1c), PPAR-α (peroxisome proliferator-activated receptor alpha), *CYP2E1* (cytochrome P450 2E1), and *NR4A1* (nuclear receptor subfamily four group A member 1) was evaluated in liver tissues from the experimental groups. These genes collectively regulate crucial metabolic pathways, including lipid metabolism, fatty acid oxidation, energy homeostasis, oxidative stress response, and inflammation. Total RNA was extracted from homogenized liver samples using TRIzol™ Reagent (Invitrogen, Thermo Fisher Scientific), followed by DNase I treatment and purification with QIAGEN columns. RNA purity was assessed using a NanoDrop™ One UV–Vis spectrophotometer (Thermo Scientific, USA). Complementary DNA (cDNA) was synthesized from the purified RNA for quantitative PCR (qPCR) using the QuantiTect^®^ SYBR^®^ Green PCR kit on a Rotor-Gene Q instrument (Qiagen, Germany). Primer efficiency was validated through standard curves generated from serial cDNA dilutions, showing efficiencies between 95% and 104% with *R* ^2^ values ≥0.98. Specific amplification and absence of primer-dimers were confirmed via melt curve analysis and agarose gel electrophoresis, and no-template controls were included to check for contamination; primer sequences are listed in Supplementary Table 1. Real-time PCR was performed using the QuantStudio™ 6 Flex Real-Time PCR System (Applied Biosystems). Gene expression levels were normalized to the Glyceraldehyde-3-Phosphate Dehydrogenase (*GABDH*), selected from four reference genes based on stability analysis using the geNorm algorithm (Vandesompele et al., 2002). Relative expression was calculated according to the method described by Schmittgen and Livak (2008), incorporating primer efficiencies in the analysis.

**TABLE 1:** Effect of Polystyrene microplastics (PS-MPs) (10 mg/kg b.wt) and Tau (200 mg/kg b.wt) oral dosing on hepatic enzymes, bilirubin, and lipid profile of adult male Swiss mice for 60 days.

Estimated parameters	Control	Tau	PS-MPs	PS-MPs + Tau
AST (U/L)	55.28 ± 3.05	41.72 ± 1.13^***^	143.0 ± 5.63^***^	92.59 ± 4.18^***/€€€^
ALT (U/L)	37.19 ± 3.60	23.74 ± 2.02^ns^	120.5 ± 4.41^***^	66.47 ± 3.69^***/€€€^
GGT (U/L)	1.52 ± 0.16	1.87 ± 0.26^ns^	9.95 ± 0.48^***^	4.45 ± 0.38^***/€€€^
Total bilirubin (mg/dL)	0.45 ± 0.02	0.41 ± 0.005^ns^	0.89 ± 0.03^***^	0.66 ± 0.032^***/€€€^
Direct bilirubin (mg/dL)	0.11 ± 0.02	0.09 ± 0.003^ns^	0.21 ± 0.05^***^	0.14 ± 0.03^***/€€€^
Indirect bilirubin (mg/dL)	0.37 ± 0.16	0.35 ± 0.012^ns^	0.69 ± 0.03^***^	0.50 ± 0.024^**/€€€^
TC (mg/dL)	113.2 ± 6.00	92.70 ± 1.80^ns^	280.3 ± 14.28^***^	179.6 ± 7.23^***/€€€^
TG (mg/dL)	90.26 ± 1.69	75.51 ± 1.58^ns^	162.5 ± 3.24^***^	130.2 ± 7.65^***/€€€^
HDLC (mg/dL)	43.51 ± 0.46	40.82 ± 0.41^ns^	55.37 ± 2.64^***^	46.25 ± 0.76 ns/_€€_
LDLC (mg/dL)	56.18 ± 2.59	58.49 ± 0.56^ns^	188.8 ± 7.89^***^	113.5 ± 10.59^***/€€€^
VLDLC (mg/dL)	20.50 ± 1.07	22.23 ± 1.87^ns^	33.50 ± 0.78^***^	28.92 ± 0.46^***/€€€^

AST, aspartate aminotransferase; ALT, alanine aminotransferase; ALP, alkaline phosphatase; TC, total cholesterol; TG, triglyceride; TG LDL, low-density lipoprotein; VLDL, Very low-density lipoprotein; HDL, High-density lipoprotein. Means within the same row carrying different superscripts are significantly different at *p* < 0.05. The values shown are means ± SE. n = 10. *P < 0.05 vs. control.

^€^ P < 0.05 vs. PS-MPs.

### 2.13 Histopathological evaluations

The specimens were then processed using the routine paraffin embedding method. Tissue sections, each 4 µm thick, were stained with Hematoxylin and Eosin (H&E) according to the protocol described by Bancroft and Layton (2013). Hepatic lesions were evaluated using a semiquantitative scoring system based on the criteria of Gibson-Corley et al. (2013). Ten randomly selected fields at ×40 magnification (Olympus BX53, Leica Application Suite) were assessed, and the scores were averaged. The evaluation was performed in a blinded manner using the following scale: 0 = normal; 1 = ≤25% affected area; 2 = 26–50%; 3 = 51–75%; and 4 = 76–100%.

### 2.14 Data analysis

Prior to statistical evaluation, the dataset was subjected to preliminary assessments to verify the assumptions of normality and homogeneity. The Shapiro-Wilk test was employed to assess the normal distribution of the data, while Levene’s test was used to examine the equality of variances. Data were presented as mean ± standard error of the mean (SEM). Statistical analyses were performed using GraphPad Prism^®^ version 7 (GraphPad Software Inc., San Diego, CA, USA). One-way analysis of variance (ANOVA) was conducted to detect group differences, and *post hoc* comparisons were made using Tukey’s multiple comparison test. A p-value of less than 0.05 was considered statistically significant. Standardized mean differences (Hedges’ g) with 95% CIs were also calculated for final body weight, liver weight, and HSI and visualized using a Forest plot.

## 3 Results

### 3.1 Characterization of PS-MP

**FIGURE 1:**
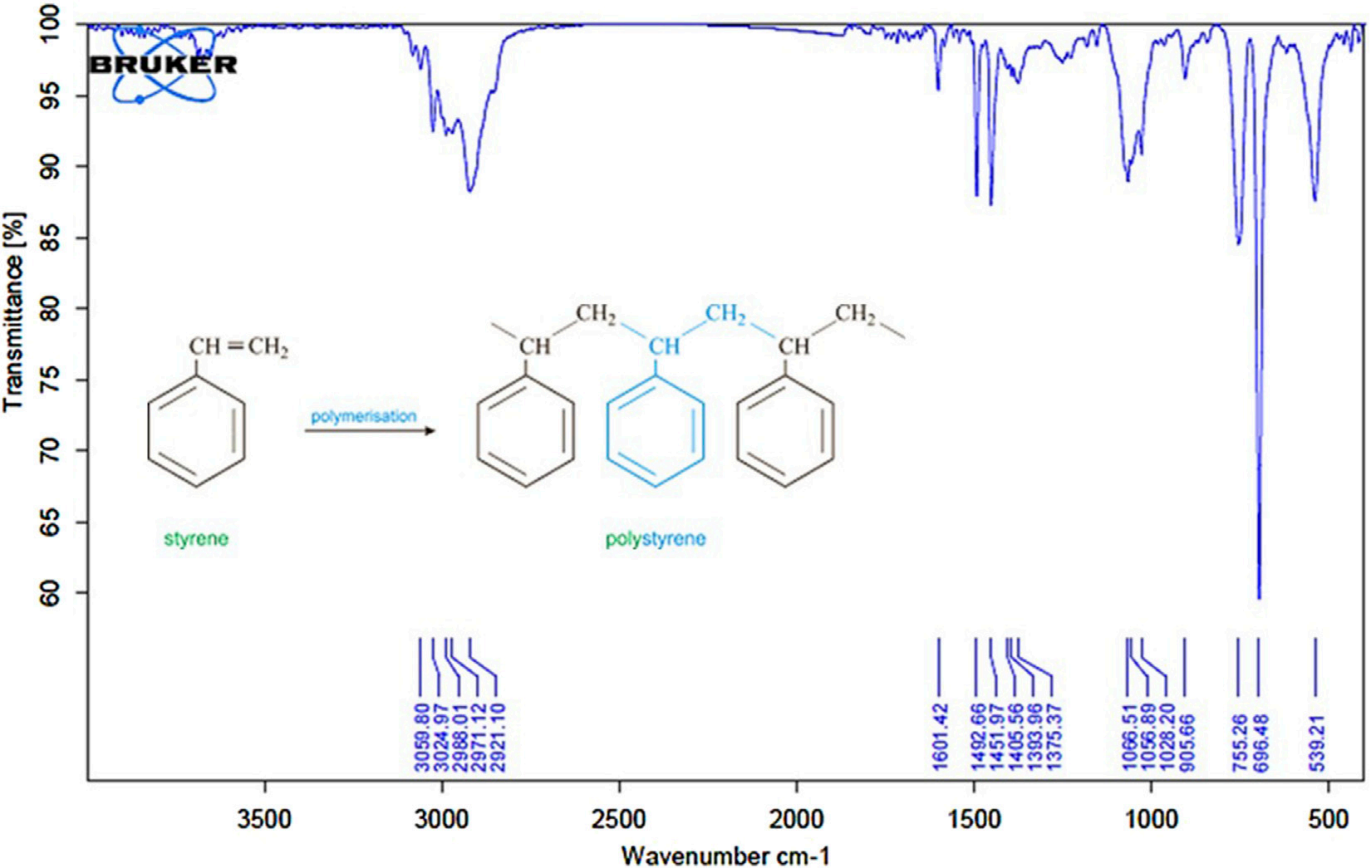
ATR‐FTIR spectrum of synthesized polystyrene microplastics (PS‐MPs). The spectrum confirms the characteristic functional groups of polystyrene.

ATR-FTIR analysis (Figure 1) confirmed the chemical structure of the synthesized PS-MPs. The spectrum showed characteristic peaks at 3,059 and 3,024 cm^-1^, corresponding to aromatic C–H stretching vibrations of the benzene ring. Additional peaks at 2,988 and 2,921 cm^1^ were assigned to aliphatic C–H and CH_2_ stretching. Peaks at 1,601, 1,492, and 1,451 cm^-1^ indicated C=C aromatic ring vibrations. Absorption bands at 755 and 696 cm^-1^ were attributed to out-of-plane C–H bending, consistent with monosubstituted aromatic rings. The absence of a peak between 1,640 and 1,680 cm^-1^ confirmed the full consumption of vinyl double bonds, indicating complete polymerization.

**FIGURE 2:**
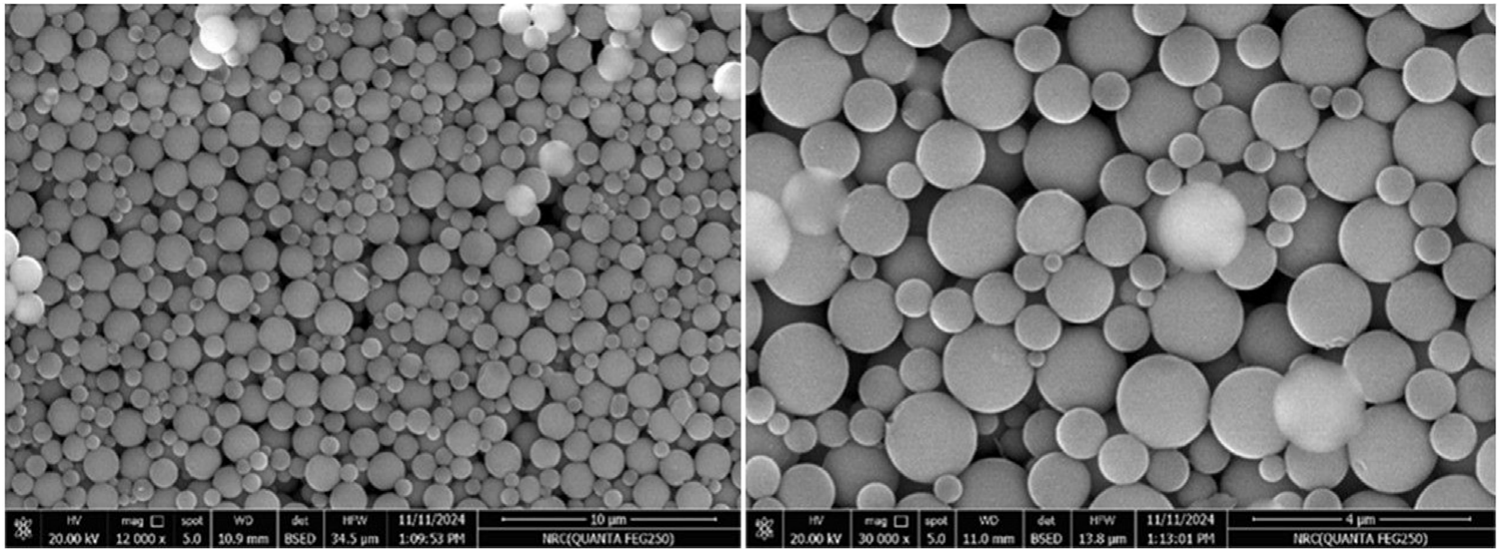
Scanning Electron Microscope (SEM) image of synthesized polystyrene micro-plastic (PS‐MPs) at two different magnifications.

Scanning Electron Microscopy (Figure 2) revealed that the synthesized particles were predominantly spherical with smooth surfaces and a narrow size distribution. The average particle diameter was approximately 1.8 µm.

**FIGURE 3:**
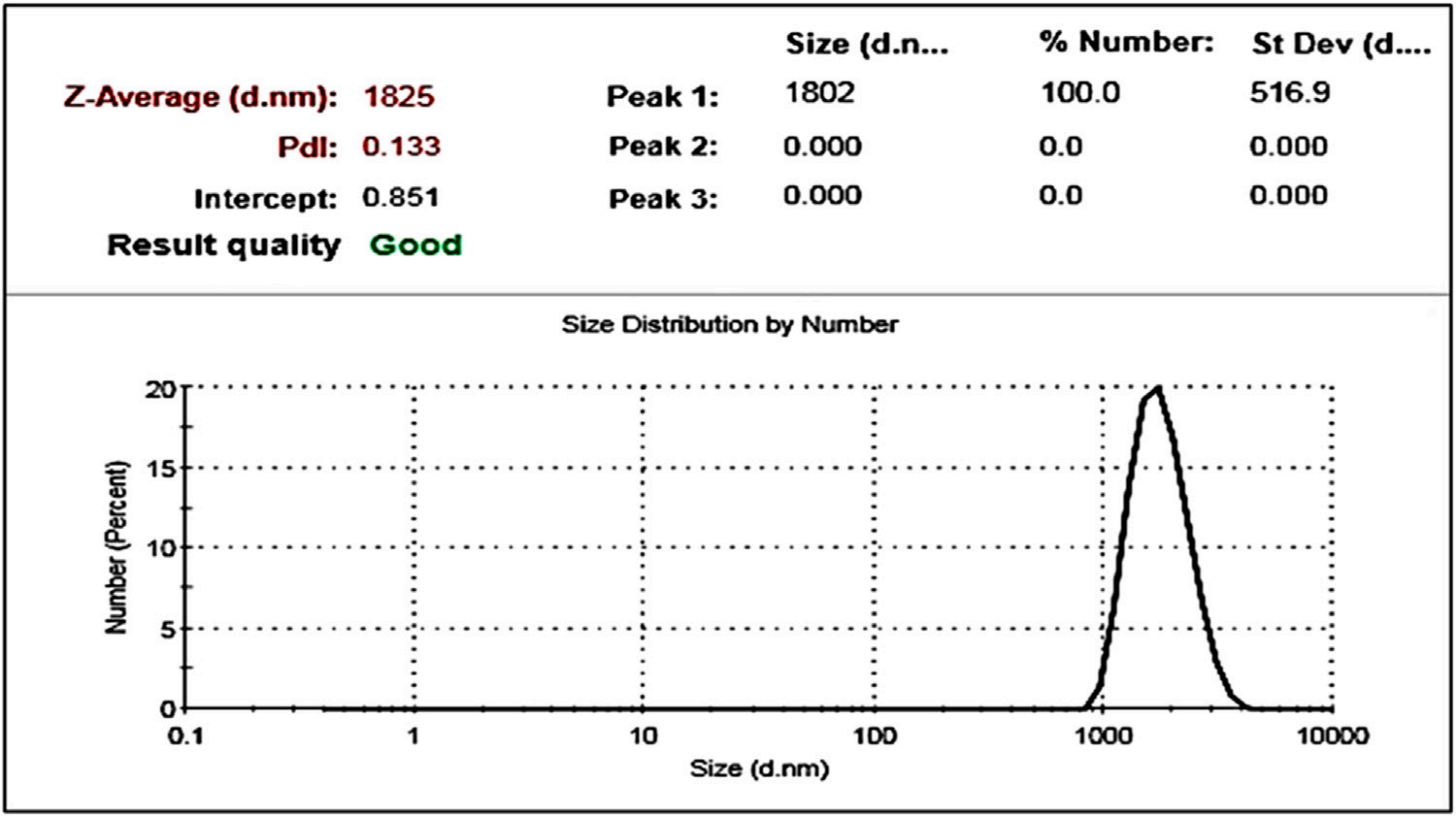
Particle size distribution of synthesized polystyrene microplastics (PS‐MPs) as determined by Dynamic Light Scattering (DLS).

Dynamic Light Scattering (DLS) (Figure 3) analysis supported the Scanning Electron Microscopy findings, confirming an average particle size of 1.8 µm. The particles displayed a low polydispersity index (PDI = 0.133), indicating a uniform size distribution.

### 3.2 Body weight and hepatosomatic index

**FIGURE 4:**
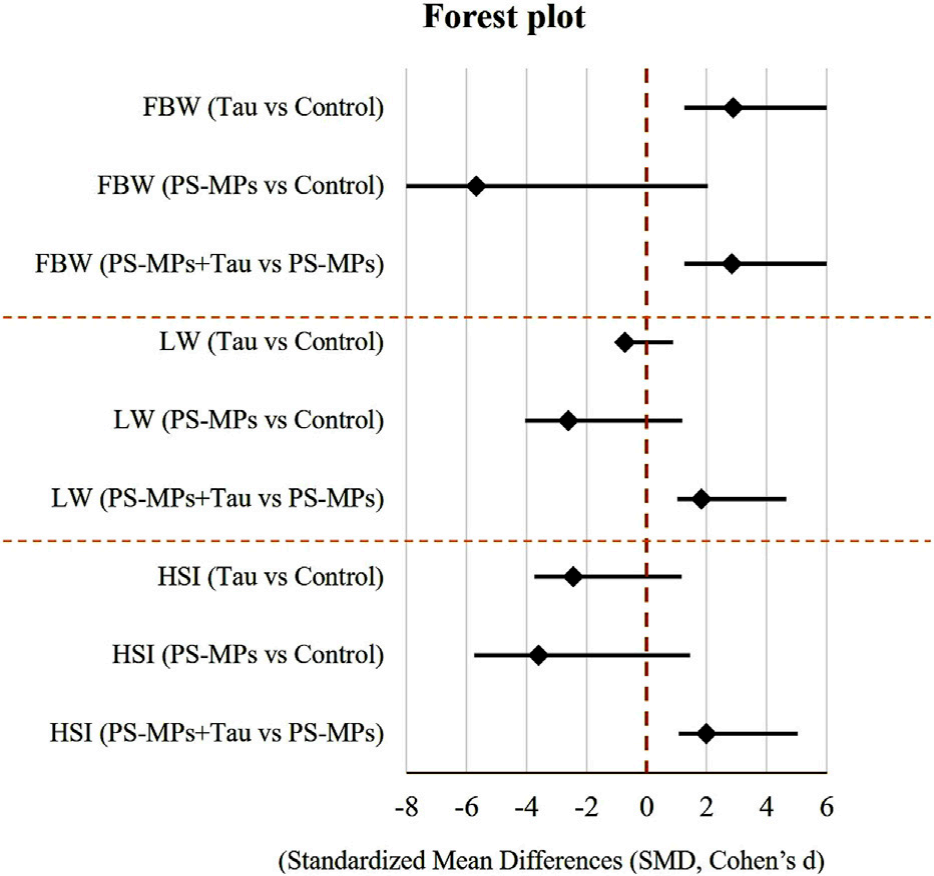
Forest plot of standardized mean differences (Hedges’ g) with 95% confidence intervals for the effects of taurine (Tau) and polystyrene microplastics (PS‐MPs) on final body weight (FBW), liver weight (LW), and hepatosomatic index (HSI) on adult male Swiss mice after 60 days of oral dosing. A vertical dashed line at 0 indicates no effect relative to Control. Negative values indicate a reduction compared to Control, while positive values indicate an increase.

Figure 4 illustrates the impact of oral exposure to PS-MPs; 10 mg/kg b. wt) and taurine (Tau; 200 mg/kg b. wt) on final body weight, liver weight, and hepatosomatic index (HSI) in adult male Swiss mice over a period of 60 days. Compared to the control group, PS-MPs exposure resulted in a 9.7% reduction in final body weight, while co-administration with Tau improved it by 6.4% relative to the PS-MPs group, indicating partial recovery.

Liver weight was reduced by 19.3% in the PS-MPs group compared to the control. However, in the co-treated group, liver weight increased by 10.5% compared to the PS-MPs group, suggesting a protective role of Tau.

Regarding the hepatosomatic index, PS-MPs caused a 27.1% decrease compared to the control group. Tau co-treatment improved the index by 15.0% relative to the PS-MPs group, reflecting a mitigating effect of Tau against PS-MP-induced hepatic impairment.

### 3.3 Hepatic enzymes, lipid, and bilirubin profile

Exposure to PS-MPs significantly disrupted liver function and lipid metabolism in adult male Swiss mice after 60 days of oral administration. Compared to the control group (Table 1), PS-MPs significantly (*P* < 0.001) elevated serum levels of AST, ALT, and GGT by approximately 159%, 224%, and more than 6.5-fold, respectively, indicating severe hepatocellular injury. Co-administration of Tau notably mitigated these elevations, reducing AST by 35%, ALT by 45%, and GGT by 55% compared to the PS-MPs group (*P* < 0.001), suggesting a potent hepatoprotective effect. Interestingly, Tau alone resulted in a significant (*P* < 0.001) 24% decrease in AST activity relative to control, reflecting its baseline liver-protective potential.

The presented data in Table 1, PS-MPs induced a significant (*P* < 0.001) increase in total, direct, and indirect bilirubin levels by 98%, 91%, and 86%, respectively, indicating impaired hepatic clearance or conjugation. Tau co-treatment improved these alterations, lowering total bilirubin by 26%, direct bilirubin by 33%, and indirect bilirubin by 28% compared to the PS-MPs group, pointing to Tau’s ability to restore hepatic excretory function. Lipid profile parameters were also adversely affected by PS-MPs exposure. Total cholesterol (TC) and triglyceride (TG) levels rose by 147% and ∼80%, respectively, while low-density lipoprotein cholesterol (LDL-C) increased dramatically by 236%. Although high-density lipoprotein cholesterol (HDL-C) increased by 27%, this likely reflects a compensatory response rather than a beneficial effect. Tau co-administration significantly reduced TC, TG, and LDL-C levels by 36%, 20%, and 40%, respectively, when compared to the PS-MPs group, indicating notable correction of dyslipidemia. Moreover, very low-density lipoprotein cholesterol (VLDL-C) levels, which were elevated by 63% in the PS-MPs group, were decreased by 14% following Tau treatment. The Tau-only group exhibited a moderate 18% reduction in TC compared to the control, with no significant effects on the other lipid fractions. PS-MPs induce pronounced liver damage and lipid abnormalities, while Tau offers considerable protection against such disturbances.

### 3.4 Oxidative stress and antioxidants

**FIGURE 5:**
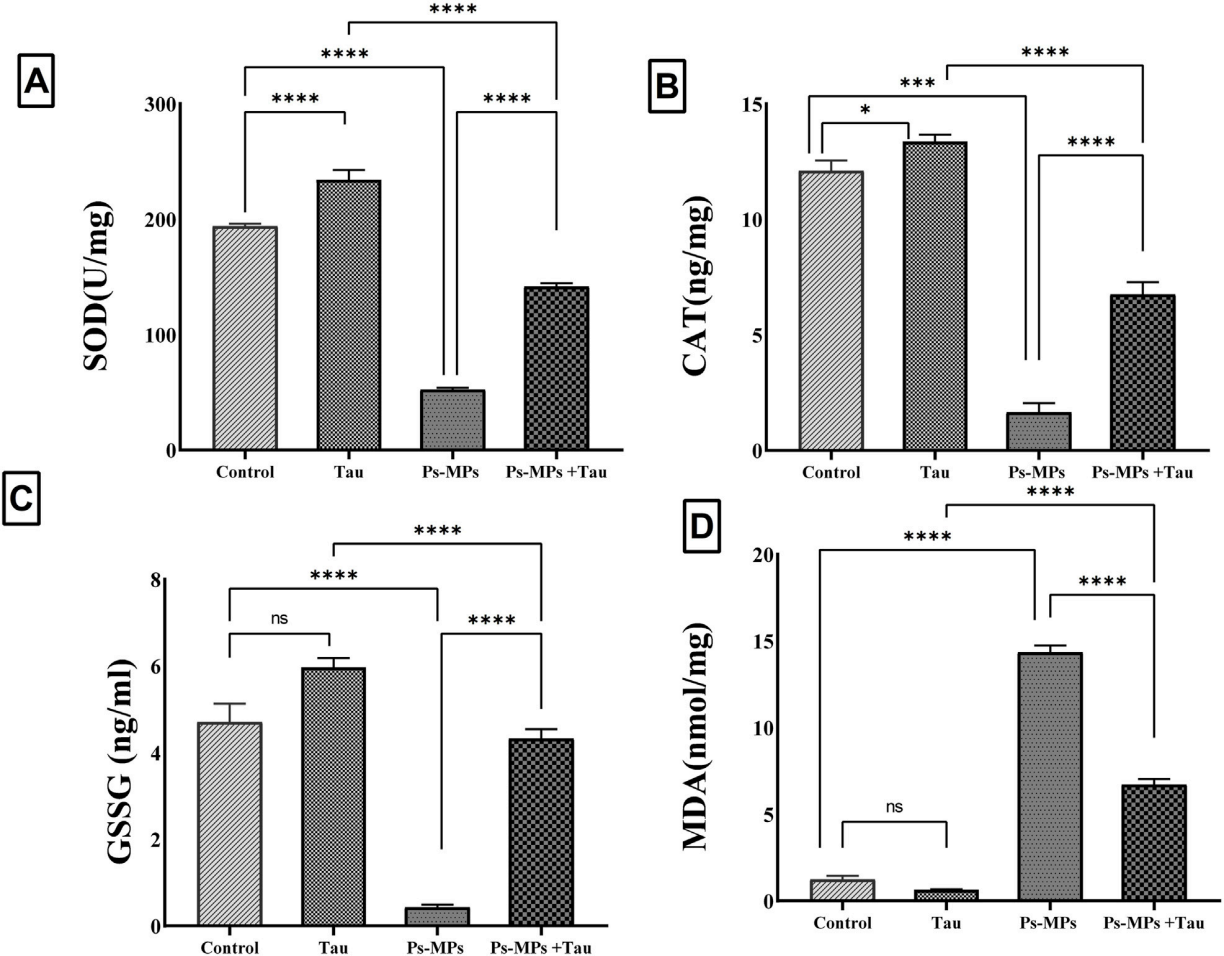
Effect of polystyrene microplastics (PS‐MPs) and/or Taurine (Tau) exposure on hepatic tissue levels of Superoxide dismutase (SOD) **(A)**, Catalase (CAT) **(B)**, Glutathione disulfide (GSSG) **(C)**, and Malondialdehyde (MDA) **(D)** in a mouse model. Data expressed as mean ± SE, n = 10 for each group. *P<0.05, ***P < 0.0001, and “ns” means “non‐significant”.

Exposure to PS-MPs led to a significant impairment in antioxidant defense and a pronounced increase in oxidative stress markers in liver tissues. SOD activity showed a significant decrease (*P* < 0.001) of approximately 87% in the PS-MPs group compared to the control, reflecting severe oxidative burden. Co-administration of Tau significantly (*P* < 0.001) restored SOD activity, increasing it by around 4.5-fold compared to the PS-MPs group, although it remained lower than the control. Tau alone also significantly enhanced SOD activity by ∼17% compared to the control. CAT activity was also markedly (*P* < 0.001) reduced in the PS-MPs group, showing an approximate 89% decrease relative to the control. Tau co-treatment substantially improved CAT activity, with a more than 4.5-fold increase compared to the PS-MPs group (Figure 5). While still below the control level, this increase signifies partial recovery of the enzymatic antioxidant capacity. The Tau group demonstrated a modest but statistically significant increase (13%) in CAT activity relative to controls.

For glutathione disulfide (GSSG), a marker of oxidative glutathione consumption, PS-MPs exposure resulted in a sharp 90% reduction compared to controls, indicating severe oxidative depletion. Tau administration alongside PS-MPs significantly (*P* < 0.001) restored GSSG levels, increasing them by approximately 7.3-fold versus the PS-MPs group (Figure 5). Tau alone induced a slight, non-significant (*P* > 0.05) increase in GSSG relative to control. Malondialdehyde (MDA), was profoundly elevated (*P* < 0.001) in the PS-MPs group, showing a more than 10-fold increase compared to control animals, reflecting intense membrane oxidative damage. Tau co-treatment significantly attenuated MDA levels by over 60% compared to the PS-MPs group.

### 3.5 Hepatic gene expression alteration and inter-group comparisons

**FIGURE 6:**
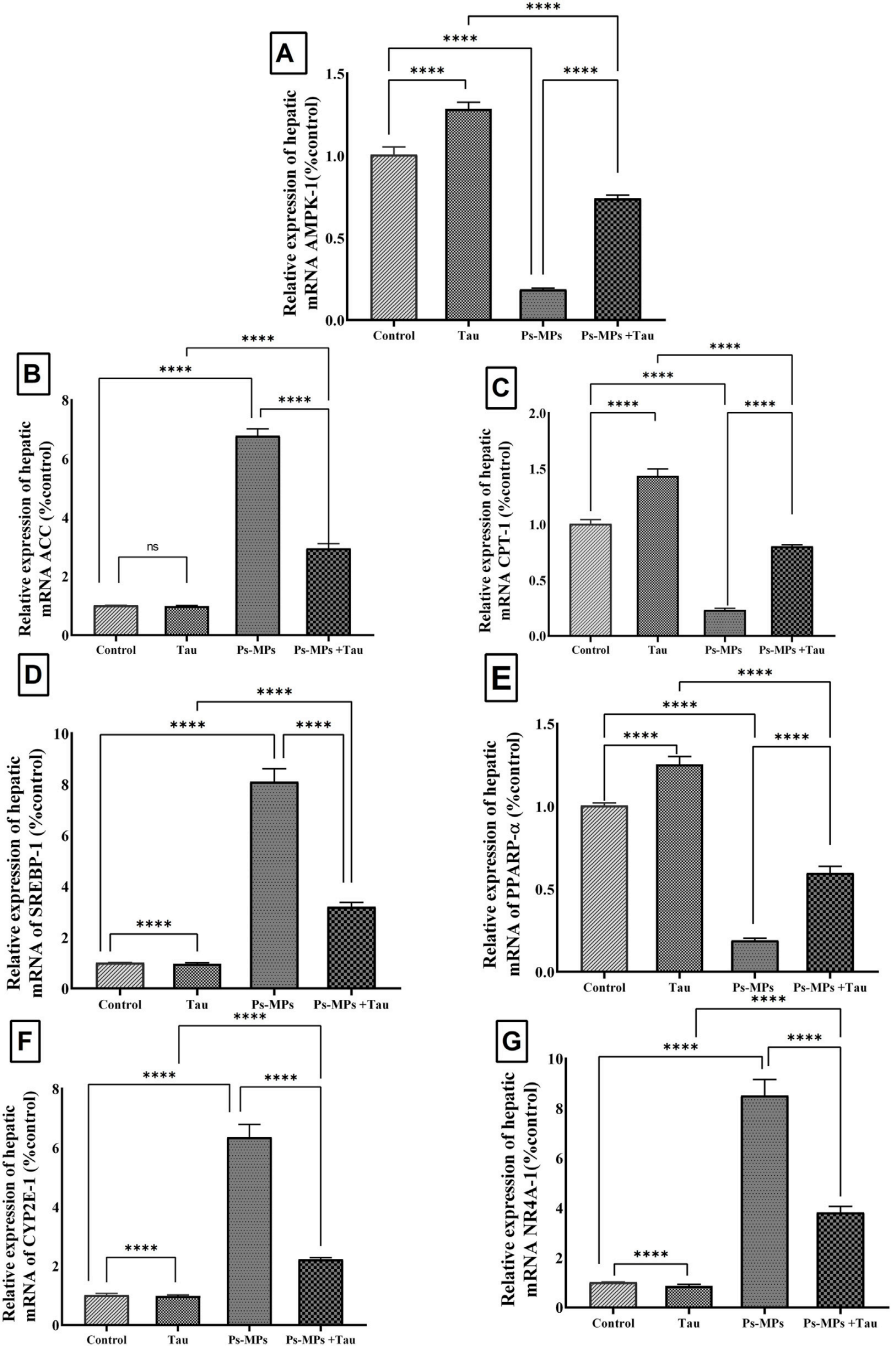
Effect of polystyrene micro-plastic (PS‐MPs) and/or Taurine (Tau) exposure on hepatic mRNA expression of AMPK‐1 (AMP‐activated protein kinase alpha-1) **(A)**, ACC (acetyl‐CoA carboxylase) **(B)**, CPT‐1 (carnitine palmitoyltransferase‐1) **(C)**, SREBP‐1 (sterol regulatory element-binding protein‐1c) **(D)**, PPAR‐α (peroxisome proliferator-activated receptor alpha) **(E)**, CYP2E1 (cytochrome P450 2E1) **(F)**, and NR4A1 (nuclear receptor subfamily 4 group A member 1)**(G)** against the normalizing gene GAPDH: glyceraldehyde‐3‐phosphate dehydrogenase. Data expressed as mean ± SE, n = 10 for each group. ****P < 0.0001, and *ns“ means ”non“significant”.

The hepatic expression of *AMPK-1* (an energy sensor that regulates cellular energy homeostasis) mRNA was significantly (*P* < 0.0001) altered following the various treatments. Exposure to PS-MPs led to a marked downregulation in *AMPK-1* expression by approximately 82.2% compared to the control group. In contrast, Tau administration alone resulted in an upregulation of *AMPK-1* by 33.3%. Notably, co-administration of Tau with PS-MPs significantly (*P* < 0.0001) restored AMPK-1 expression, with an increase of approximately 362.5% relative to the PS-MPs group (Figure 6).

Expression levels of *ACC* mRNA, a key enzyme in fatty acid biosynthesis, remained statistically unchanged in the Tau group compared to the control. However, a significant upregulation of 5.02-fold was observed in the PS-MPs group. Co-treatment with Tau significantly attenuated this increase, reducing *ACC* expression by 58.5% compared to the PS-MPs group (Figure 6).

The expression of *CPT-1* mRNA (Figure 6), which is involved in mitochondrial fatty acid oxidation, was elevated by 50% in the Tau group versus the control. In contrast, PS-MPs exposure suppressed *CPT-1* expression by 75% versus control (*P* < 0.0001), while Tau co-administration restored its expression by 1.7-fold compared to the PS-MPs group.

A substantial increase (*P* < 0.0001) in *SREBP-1* mRNA expression, approximately 6-fold, was detected in the PS-MPs group compared to the control, suggesting enhanced lipogenesis. Tau alone caused only a minor, non-significant (*P* > 0.05) increase of 22.2%. Co-treatment with Tau markedly decreased the PS-MP-induced upregulation by 46.6%, indicating a mitigating effect.

Expression of *PPAR-α* mRNA, a key regulator of lipid catabolism, was upregulated by 27.3% in the Tau group, whereas PS-MPs caused a significant suppression by 76.4% compared to the control. Tau co-administration significantly counteracted this suppression, resulting in a 154.5% increase relative to the PS-MPs group.

The oxidative stress marker *CYP2E1* mRNA was dramatically upregulated by 4.5-fold in the PS-MPs group. Tau supplementation in combination with PS-MPs significantly reduced this expression by 53.6%, suggesting a protective effect. Tau alone slightly downregulated *CYP2E1* by 11.7% compared to control.

Finally, *Nrf4-A1* mRNA expression was elevated by approximately 7.5-fold following PS-MPs exposure. Tau co-administration significantly reduced this level by 54.2% compared to PS-MPs alone, while taurine alone caused a minor downregulation of 17.4% versus control.

### 3.6 Histopathological alterations of the mouse liver in different experimental groups

**FIGURE 7:**
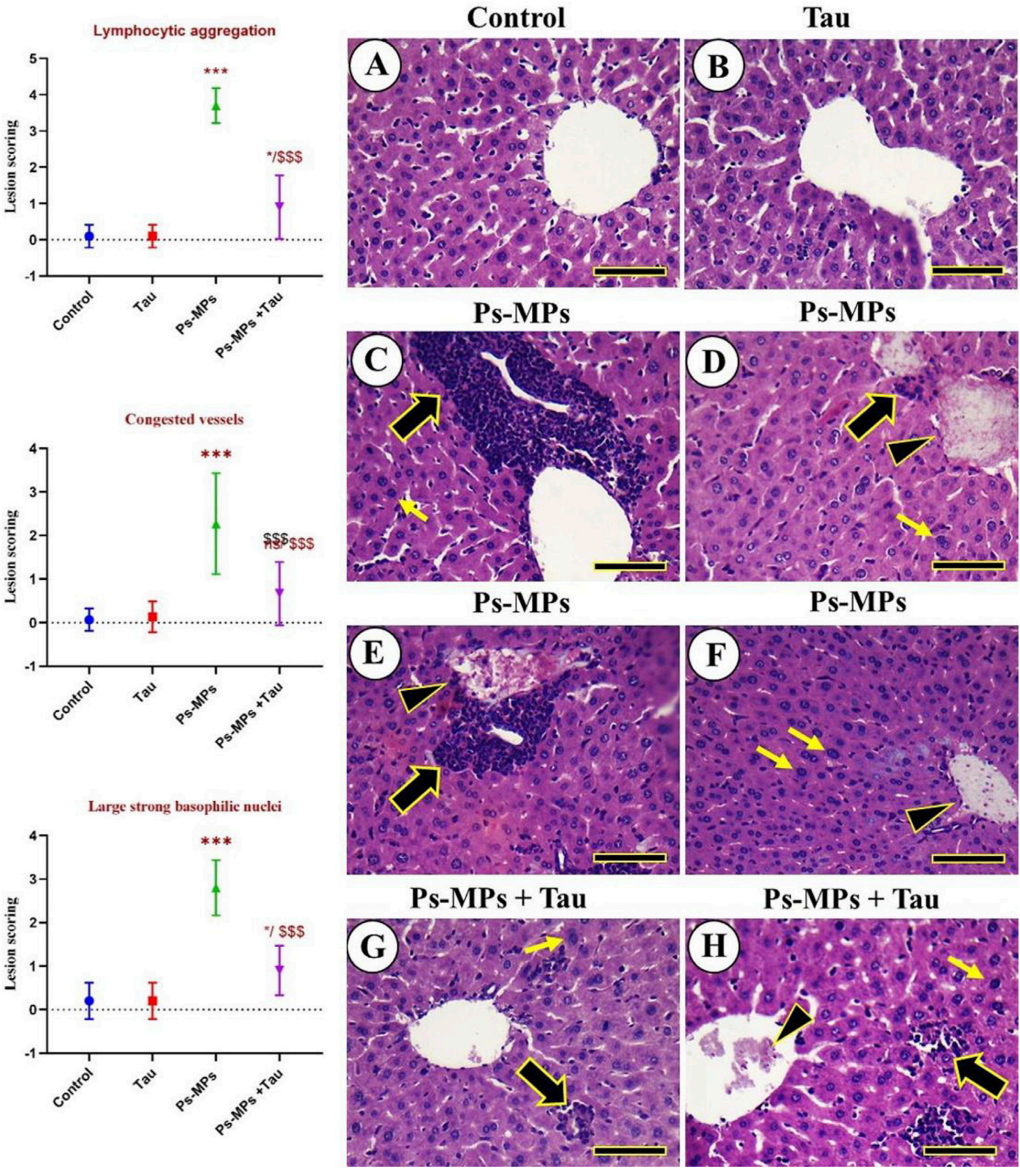
Histopathological examination of the liver of mice. **(A)** Negative control. **(B)** Tau. **(C–F)** Ps‐MPs group revealing congested central and portal veins, massive aggregations of lymphocytic cells, and the hepatocytes appeared markedly swollen, with a disorganized arrangement, and exhibited strong, large nuclei with strong basophilic staining. **(G,H)** Ps‐MPs + Tau group. Congested veins (arrowheads), aggregated lymphocytic aggregations (thick black arrows), and large nuclei with strong basophilic staining (thin yellow arrows). Scale bar =50 mm. Moreover, the figure explains the lesion scoring in the hepatic tissue of mice in all experimental groups in the form of a histogram, which indicates the different lesion scoring, including Lymphocytic aggregation, Congested vessels, and large, strong basophilic nuclei.

No histopathological hepatic lesions were observed in the negative control and Tau groups, and the hepatocytes displayed a healthy cord-like arrangement (Figures 7A,B). In contrast, liver samples from the PS-MPs group showed congested central and portal veins, massive lymphocytic infiltrations, and hepatocytes that appeared markedly swollen, disorganized, and contained large nuclei with intense basophilic staining (Figures 7C– F). Mice treated with PS-MPs + Tau exhibited an improved hepatic architecture compared to the PS-MPs group (Figure 7E).

The scoring results demonstrated significant hepatic alterations in the PS-MPs group compared to the control and Tau groups. Lymphocytic aggregation in the PS-MPs group exhibited a marked increase in lymphocytic infiltration compared to the control and Tau groups (***p < 0.001). In the PS-MPs + Tau group, lymphocytic aggregation was significantly reduced compared to the PS-MPs group *(###p* < 0.001), although it remained elevated relative to controls. Severe vascular congestion was observed in the PS-MPs group, with lesion scores significantly higher than in the control and Tau groups (***p < 0.001). Co-administration with Tau significantly alleviated vascular congestion *(###p* < 0.001 vs PS-MPs), though not to control levels. Lastly, regarding the Large basophilic nuclei Hepatocytes with enlarged, strongly basophilic nuclei were significantly more frequent in the PS-MPs group compared to the control and Tau groups (**p < 0.01). In contrast, Tau supplementation markedly attenuated this alteration vs. PS-MPs, suggesting a protective effect.

## 4 Discussion

The global production of plastic has risen dramatically over recent decades, resulting in plastic pollution becoming a critical environmental issue worldwide (Chen et al., 2021; Horton et al., 2017). PS-MPs are among the most commonly used polymers in personal care products and are also prevalent in marine environments (Akanyange et al., 2022). Furthermore, PS-MPs rank among the most frequently identified microplastic types in food, drinking water, and other beverages (Vitali et al., 2023). Following their entry into the circulatory system, microplastics are largely transported to and processed by the liver and kidneys (Goodman et al., 2022). Consequently, the liver is recognized as a key target organ for microplastic-related toxicity. These particles can reach the liver via several routes, including gastrointestinal absorption, penetration through the skin, or direct transport through the bloodstream (Ma et al., 2021). Studies have indicated that the accumulation of microplastics within hepatic tissue can initiate autophagy and programmed cell death (apoptosis) in liver cells (Lu et al., 2016). Moreover, such accumulation can interfere with lipid metabolic processes, exacerbate hepatic fibrosis, and play a role in the development of non-alcoholic fatty liver disease (NAFLD) (Auguet et al., 2022; Lai et al., 2021).

### 4.1 Body weight and hepatosomatic index

In the present study, exposure to PS-MPs (10 mg/kg b. wt) led to reductions in body weight, liver weight, and HSI by the end of the experimental period compared to the control group. These findings are in agreement with those reported by Lu et al. (2018). Conversely, a study by Huang et al. (2023) demonstrated that mice exposed to relatively low doses of microplastics over a 10-week period became overweight. This was attributed to enhanced appetite and increased feed intake, likely driven by altered lipid metabolism and reduced physical activity. However, the same study reported the opposite effect at higher microplastic doses (100 μg/mL), where exposed mice exhibited significant declines in body weight relative to controls. Furthermore, it has been previously found that PS-MP exposure leads to gut microbiota dysbiosis, intestinal inflammation, and impaired barrier integrity, which reduce nutrient absorption and caloric uptake (Xu et al., 2024). These processes damage hepatocytes, contributing to cell death and reduced liver mass, thereby lowering HSI. Furthermore, our results indicated that PS-MPs played a role in the alteration of lipid metabolism and lipid profile detected in the exposed group.

Yet, our findings revealed that co-treatment with Tau could elevate the body weight, liver weight, and HSI when it is administered with PS-MPS for 60 days. This is a lightened point which proves that Tau could be a candidate for supporting health issues correlated with PS-MPs administration and toxicity. Tau has been shown to enhance energy metabolism and glucose tolerance by improving insulin sensitivity and glucose uptake in the liver and peripheral tissues. One mechanism involves increased phosphorylation of Akt (protein kinase B), a central regulator in the insulin signalling pathway. Enhanced Akt activity promotes glucose uptake, glycogen synthesis, and lipid metabolism, which collectively contribute to better energy utilization and reduced fat accumulation.

Importantly, improved glucose handling and metabolic efficiency reduce the metabolic burden on the liver, preventing hepatic steatosis and maintaining overall body weight (Musso et al., 2012). Thus, Tau’s ability to improve liver glucose control even more so in the Tau group, combined with PS-MPs, directly supports the restoration of body weight, as it helps reverse or prevent the metabolic dysregulation (e.g., insulin resistance, altered lipid storage) often caused by microplastic exposure.

### 4.2 Liver enzymes and bilirubin profile

In the present study, oral exposure to PS-MPs led to significant elevations in serum levels of aspartate aminotransferase (AST) and alanine aminotransferase (ALT), which are well-established biomarkers of hepatocellular injury. The marked increase in these enzymes suggests compromised hepatocyte membrane integrity and leakage of intracellular contents into circulation, reflecting liver dysfunction (Hatipoglu et al., 2024). These findings align with those reported by Mu et al. (2022), who demonstrated PS-MPs-induced hepatotoxicity in mice, likely mediated by oxidative stress and inflammatory responses, which is indicated in the current study by suppression of antioxidants, enhanced lipid peroxidation, and upregulated expression of inflammatory genes. Furthermore, the current data showed a concurrent rise in total, direct (conjugated), and indirect (unconjugated) bilirubin levels in the PS-MPs-exposed group. Elevated bilirubin, particularly the direct fraction, often indicates hepatocellular damage or impaired bilirubin excretion, while an increase in indirect bilirubin may suggest disruptions in hepatic uptake or conjugation processes (Alqahtani et al., 2023).

### 4.3 Oxidative stress

In the current study, exposure to PS-MPs induced marked oxidative stress in the liver, as demonstrated by a significant decrease in the activities of key antioxidant enzymes, including SOD and CAT, and a notable increase in MDA levels, a marker of lipid peroxidation. In addition, GSSG levels were significantly reduced in the PS-MPs group, indicating impaired redox homeostasis. These findings are consistent with previous studies, such as Wan et al. (2019) and Wang et al. (2022), which reported decreased antioxidant capacity and elevated oxidative damage following PS-MPs exposure in zebrafish and mudskipper larvae, respectively. Although Deng et al. (2017) observed increased SOD and GSH-Px activity with decreased CAT in mice, such discrepancies across studies have been attributed to differences in particle size, concentration, exposure duration, and species or tissue-specific responses, as suggested by Prokić et al. (2019).

In our study, co-treatment with Tau significantly restored antioxidant enzyme activities and reduced MDA levels compared to the PS-MPs-only group. This antioxidant effect is likely linked to Tau’s well-documented capacity to scavenge ROS, stabilize cellular membranes, and upregulate endogenous antioxidant defenses (Surai et al., 2021). Tau has been shown to enhance mitochondrial function and reduce oxidative injury in various models (Baliou et al., 2021), including hepatic tissue, thereby mitigating PS-MPs-induced oxidative damage. Tau is found to counteract oxidative stress damage in liver tissue in several previous studies.

### 4.4 Gene expression

Our findings demonstrate that exposure to PS-MPs leads to significant dysregulation of genes involved in hepatic energy metabolism, lipid homeostasis, and oxidative stress, with clear alterations in the *AMPK/NR4A1* signalling axis. As shown in the current study (Figure 5), PS-MPs exposure markedly downregulated hepatic *AMPK-1, CPT-1, and PPAR-α* expression, while significantly upregulating *ACC, SREBP-1, CYP2E1, and NR4A1*. These changes suggest suppressed fatty acid oxidation, enhanced lipogenesis, and increased oxidative burden. The published study supports this mechanistic insight, showing that PS-MPs activate *NR4A1* (Chiu et al., 2025), a nuclear receptor that acts upstream of *AMPK* signalling, contributing to an energy metabolism imbalance and hepatic steatosis. In contrast to expected *AMPK* activation in energy-stressed cells (Feng et al., 2024). Our results show reduced *AMPK-1* expression in PS-MPs-exposed mice livers, possibly reflecting a disrupted or maladaptive *NR4A1-AMPK* signalling loop. The increased *ACC and SREBP-1* promote lipogenesis (Li et al., 2014), while reduced *CPT-1* and *PPAR-α* expression impairs mitochondrial fatty acid β-oxidation (Hardwick et al., 2009), further exacerbating lipid accumulation. Elevated *CYP2E1*, a ROS-generating enzyme, and upregulated *NR4A1* expression suggest oxidative stress and pro-inflammatory responses (Ma et al., 2024). Importantly, co-administration of taurine significantly reversed most of these gene expression changes, restoring *AMPK-1, PPAR-α, and CPT-1* levels, and suppressing *NR4A1, ACC, and SREBP-1*, which implies that Tau may counteract PS-MPs-induced metabolic disruption through modulation of the *NR4A1/AMPK/PPAR-α* pathway.

In contrast to some previous studies that reported downregulation of *SREBP-1* and upregulation of lipolytic regulators such as *PPAR-α and PGC-1α,* our current findings demonstrated a marked increase in hepatic SREBP-1 expression along with a significant suppression of *PPAR-α and AMPK-1* following PS-MPs exposure. This expression pattern indicates a metabolic shift toward enhanced lipogenesis and impaired fatty acid oxidation, contributing to lipid accumulation in hepatic tissue (Garcia and Shaw, 2017). Given that AMPK is a known inhibitor of *SREBP-1* and an activator of both *PPAR-α* and *PGC-1α* (de Souza et al., 2017), the observed reduction in *AMPK-1* expression likely contributes to the upregulation of *SREBP-1* and downregulation of *PPAR-α.* These results highlight a disrupted AMPK-mediated regulatory axis in the liver following PS-MPs exposure, favouring lipid synthesis and energy imbalance.

Herein, the obtained results show that Tau exerts a significant protective effect against PS-MPs-induced hepatic gene dysregulation by modulating key metabolic and oxidative stress pathways. In the present study, Tau co-administration effectively restored the expression of *AMPK-1, PPAR-α, and CPT-1*, while suppressing *NR4A1, ACC*, and *SREBP-1*, indicating its role in rebalancing energy metabolism and lipid homeostasis.

One of the central mechanisms by which Tau exerts this effect is through activation of AMP-activated protein kinase (*AMPK*), a master regulator of cellular energy status. As reported by Morsy et al. (2021), Tau enhances *AMPK* activity, which in turn inhibits lipogenesis by downregulating *SREBP-1 and ACC*, and promotes fatty acid β-oxidation via upregulation of *PPAR-α and CPT-1.* Moreover, Tau’s antioxidant properties contribute to its regulatory role. Elevated *NR4A1 and CYP2E1* expressions, both linked to oxidative stress and inflammation, were significantly reduced following taurine treatment. Yao et al. (2009) reported that Tau decreases *CYP2E1* expression and suppresses ROS production, thereby mitigating oxidative damage. Additionally, Zhang et al. (2023) demonstrated that Tau negatively regulates *NR4A1,* potentially disrupting its pathological interaction with *AMPK* and restoring energy balance.

Through the activation of *AMPK*, enhancing fatty acid oxidation, inhibiting lipogenesis, and reducing oxidative stress, Tau effectively re-establishes a normal hepatic gene expression profile. This highlights its therapeutic potential in alleviating microplastic-induced metabolic disturbances in the liver.

### 4.5 Histopathological alterations

The histopathological findings in PS-MPs-exposed liver tissue revealed significant structural damage, including congestion of central and portal veins, lymphocytic infiltration, and marked hepatocellular swelling and disorganization, accompanied by enlarged, basophilic nuclei, hallmarks of hepatic inflammation, oxidative injury, and cellular stress. These alterations reflect the hepatotoxic potential of PS-MPs, which may disrupt vascular integrity, initiate immune responses, and impair hepatocyte morphology and function (Elsheikh et al., 2023). However, co-administration of Tau markedly alleviated these pathological features, with notable preservation of hepatic architecture and reduction in inflammatory cell infiltration and hepatocellular degeneration. These protective effects are consistent with Tau’s well-established cytoprotective, anti-inflammatory, and antioxidant properties (Liu et al., 2017).

Furthermore, Tau’s capacity to regulate intracellular calcium homeostasis and osmoregulation (El Idrissi and Trenkner, 2003; Schaffer et al., 1980) helps maintain hepatocyte structural integrity, preventing the swelling and nuclear abnormalities observed in PS-MPs-exposed tissues (de Oliveira Ramos et al., 2018).

## 5 Conclusion

This study reveals that chronic exposure to PS-MPs at an average particle diameter of approximately 1.8 µm induces significant hepatic toxicity in mice, as evidenced by disrupted liver function, oxidative stress, lipid metabolic imbalance, and altered expression of key regulatory genes. The observed hepatotoxicity was marked by elevated liver enzymes, lipid abnormalities, oxidative damage, and histopathological alterations. Notably, co-administration of Tau effectively mitigated these deleterious effects. Tau’s protective role appears to be mediated through enhancement of antioxidant defenses and restoration of energy and lipid metabolic pathways, including modulation of *AMPK-1, CPT-1, SREBP-1, PPAR-α*, and related genes. These findings support the potential of Tau as a promising therapeutic agent against microplastic-induced hepatic injury. Also, these findings underscore the possibility that chronic microplastic exposure may contribute to the growing burden of metabolic and cardiovascular diseases in humans, particularly in populations with high environmental exposure. Further research is warranted to evaluate these risks and explore taurine or related interventions as potential protective strategies in the context of human health and environmental toxicology.

## Data Availability

The original contributions presented in the study are included in the article/Supplementary Material, further inquiries can be directed to the corresponding author.
