# Supplementary material for: Palliative effect of taurine against hepatic injury induced by polystyrene microplastics through antioxidant and metabolic pathway modulation in mice
Source: Front Pharmacol. 2025 Oct 2;16:1665161. doi: 10.3389/fphar.2025.1665161 (PMC12547698; doi:10.3389/fphar.2025.1665161)
Supplement: Supplementary file 1 [file Table1.docx]

**Table 1 Supplementary .** Primer sequences, accession number, and product size for the quantitative RT-PCR for the analyzed genes in the hepatic tissue.

| **Target gene** | **forward primer** | **reverse primer** | **bp** | **accession no.** |
| --- | --- | --- | --- | --- |
| AMPK | GAAAGTGAAGGTGGGCAAGC | GATGTGAGGGTGCCTGAACA | 145 | [NM_001355640.1](https://www.ncbi.nlm.nih.gov/entrez/viewer.fcgi?db=nucleotide&id=1245684361) |
| ACC-1 | GGAGGCGGATATCTGCTGAG | ATCGGGAGTGCTGGTTTAGC | 120 | [NM_133360.3](https://www.ncbi.nlm.nih.gov/entrez/viewer.fcgi?db=nucleotide&id=2215162839) |
| CPT-1a | GTCCTGCAACTTTGTGCTGG | CAGGTGCTGGTGCTTTTCAC | 97 | [NM_013495.2](https://www.ncbi.nlm.nih.gov/entrez/viewer.fcgi?db=nucleotide&id=162287141) |
| SREbp-1 | CAGACTCACTGCTGCTGACA | GATGGTCCCTCCACTCACCA | 134 | [NM_011480.4](https://www.ncbi.nlm.nih.gov/entrez/viewer.fcgi?db=nucleotide&id=928192568) |
| PPARA | TGCCTTCCCTGTGAACTGAC | TGGGGAGAGAGGACAGATGG | 142 | [NM_001113418.1](https://www.ncbi.nlm.nih.gov/entrez/viewer.fcgi?db=nucleotide&id=164663879) |
| CYP2E1 | TGGCTACAAGGCTGTCAAGG | CGGGCCTCATTACCCTGTTT | 195 | [NM_021282.3](https://www.ncbi.nlm.nih.gov/entrez/viewer.fcgi?db=nucleotide&id=1394533585) |
| NR4A1 | GGGTGAGGGGATGTTGTGAG | ACTGACGGAGCAAACTCTGG | 106 | [NM_001411254.1](https://www.ncbi.nlm.nih.gov/entrez/viewer.fcgi?db=nucleotide&id=2288874452) |
| GAPDH | GCATCTTCTTGTGCAGTGCC | TACGGCCAAATCCGTTCACA | 74 | [NM_017008.4](https://www.ncbi.nlm.nih.gov/entrez/viewer.fcgi?db=nucleotide&id=402691727) |

AMPK-1 (AMP-activated protein kinase alpha-1), ACC (acetyl-CoA carboxylase), CPT-1 (carnitine palmitoyltransferase-1), SREBP-1 (sterol regulatory element-binding protein-1c), PPAR-α (peroxisome proliferator-activated receptor alpha), CYP2E1 (cytochrome P450 2E1), and NR4A1 (nuclear receptor subfamily 4 group A member 1) GAPDH: glyceraldehyde-3-phosphate dehydrogenase.
